# The complete chloroplast genome of *Aster sampsonii* (Hance) Hemsl, a perennial herb

**DOI:** 10.1080/23802359.2021.1961623

**Published:** 2021-08-09

**Authors:** Qinxiang Chang, Yan Li, Pengguo Xia, Xiang Chen

**Affiliations:** a Institute of Landscape, Taiyuan University, Taiyuan, China; b Key Laboratory of Plant Secondary Metabolism and Regulation of Zhejiang Province, College of Life Sciences and Medicine, Zhejiang Sci-Tech University, Hangzhou, China

**Keywords:** Chloroplast genome, *Aster sampsonii* (Hance) Hemsl, phylogenetic analyses

## Abstract

*Aster sampsonii* (Hance) Hemsl is endemic to China. The complete chloroplast genome of *Aster sampsonii* was studied here. The genome was 152,686 base pair (bp) in length, containing a large single-copy (LSC) region of 84,345 bp, a small single-copy region (SSC) of 18,249 bp and a pair of inverted repeats (IRs) of 25,046 bp. It contains 132 unique genes, including 86 protein-coding genes, 36 transfer RNA (tRNA) genes, and eight ribosomal RNA (rRNA) genes. The GC content of the complete chloroplast genome sequence was 37.3%. Phylogenetic analyses using complete chloroplast genomes showed that *Aster sampsonii* is most closely related to *Aster hypoleucus* (NC_046503.1).

*Aster* L. is the largest genus in the family Asteraceae. The genus, which contains about 500 species, is widely distributed in Asia, Europe and North America. There are about 120 species in China, which are distributed throughout the country (Zhang et al. 2021). Among them, *Aster sampsonii* is endemic to China where it is mainly distributed in Guangdong and Hunan provinces. *Aster sampsonii* usually used as an ornamental, but also has a bacteriostatic effect whose main component is terpenoids (Gao 2010). In this study, the complete chloroplast genome of *Aster sampsonii* are de novo assembled, which would provide genomic data for related studies. At the same time, a phylogenetic tree was generated to reveal its relationship with other species.

The samples of *Aster sampsonii* were collected from Ningshan County, Shannxi Province, China (33°38′1.24″N, 108°45′9.9″E, altitude of 1134 m). The voucher specimen was preserved at XBGH (The Herbarium of Xi’an Botanical Garden) (Voucher number: *Xun Lulu* et al *01326*). Total genomic DNA was extracted using the modified CTAB method (Doyle and Doyle 1987) and TruSeq DNA Sample Prep Kit (Illumina, USA) was used to construct a genomic library consisting of an insert size of 350 bp. Sequencing was carried out on an Illumina NovaSeq 6000 platform. Raw PE reads of about 2.23 Gb were trimmed and high quality PE reads of about 2.21 Gb were subjected to de novo assembly. NOVOplasty was used for the de novo assembly of chloroplast genome (Dierckxsens et al. 2017). The sequence was annotated using GeSeq (https://chlorobox.mpimp-golm.mpg.de/geseq-app.html), and visually checked in Geneious v8.0.2 (Kearse et al. 2012) using the chloroplast genome of *Aster hypoleucus* (GenBank accession number (NC_046503.1)) as reference. Finally, a complete chloroplast genome of *Aster sampsonii* was obtained and submitted to Genbank (GenBank accession number (MW929181)).

The complete chloroplast genome of *Aster sampsonii* (Hance) Hemsl (GenBank accession number: (MW929181)) was 152,686 bp in length with 37.3% GC contents, consisting of a pair of inverted repeat regions (IRa and IRb) with same length (25,046 bp) separated by the large single copy (LSC, 84,345 bp) and small single copy (SSC, 18,249 bp) regions. A total of 132 genes were identified, including 86 protein-coding genes, eight ribosomal RNA (rRNA) genes, and 37 transfer RNA (tRNA)genes.

Phylogenetic tree was constructed using IQTREE v1.6.7 (Nguyen et al. 2015) for *Aster sampsonii* and 27 related species and one outgroup chloroplast genomes, with the best selected K3Pu + F+R5 model and 1000 bootstrap replicates. The chloroplast genome of the *Aster sampsonii* showed the closest relationship to that of previously reported *Aster hypoleucus* (NCBI reference sequence ID NC_046503.1) (Figure 1). The newly characterized *Aster sampsonii* complete chloroplast genome would provide essential data for further study on the phylogeny and evolution of *Aster* and provide useful resources for better understanding the physiology and evolution of compositae. The phylogenetic tree illuminates the phylogenetic relationships among *Aster* species.

**Figure 1.**
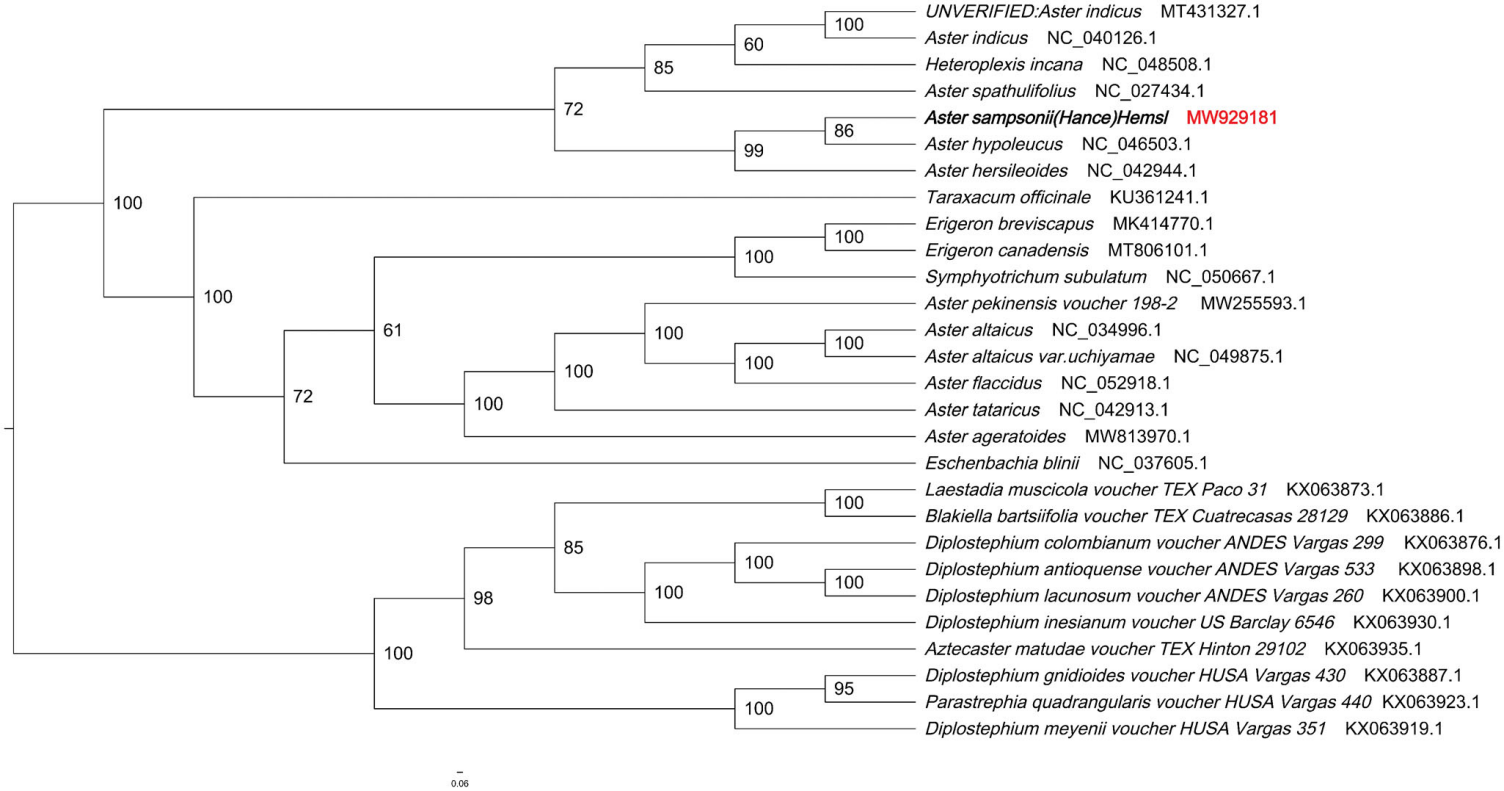
Phylogenetic tree showing the relationship between *Aster sampsonii* and 27 Compositae species. Phylogenetic tree was constructed based on the complete chloroplast genomes using maximum likelihood (ML) with 5000 bootstrap replicates. Numbers in each the node indicated the bootstrap support values.

## Data Availability

The data that support the findings of this study are openly available in NCBI (https://www.ncbi.nlm.nih.gov) GenBank with the accession number (MW929181).
